# When is a causal illusion an illusion? Separating discriminability and bias in human contingency judgements

**DOI:** 10.1177/17470218241293418

**Published:** 2024-11-19

**Authors:** Stephanie Gomes-Ng, Sarah Cowie, Douglas Elliffe

**Affiliations:** 1 Department of Psychology, Auckland University of Technology, Auckland, New Zealand; 2 School of Psychology, The University of Auckland, Auckland, New Zealand

**Keywords:** Causal illusion, signal detection, adventitious reinforcement, contingency judgement, discriminability, bias

## Abstract

Humans often behave as if unrelated events are causally related. As the name suggests, such *causal illusions* imply failures to detect the absence of a causal relation. Taking a signal detection approach, we asked whether causal illusions indeed reflect failures of discriminability, or whether they reflect a general bias to behave as if events are causally related. Participants responded in a discrete trial procedure in which point gains, point losses, or no change in points occurred dependently on or independently of responding. Participants reported whether each event was response-dependent or response-independent by choosing between two stimuli, one corresponding to reporting “I did it” and the other to “I didn’t do it.” Overall, participants responded accurately in about 80% of trials and were biased to report that events depended on responding. This bias was strongest after point gains and for higher-performing participants. Such differences in event-specific biases were not related to response rates; instead, they appear to reflect more fundamental differences in the effects of appetitive and aversive events. These findings demonstrate that people can judge causality relatively well, but are biased to attribute events to their own behaviour, particularly when those events are desirable. This highlights discriminability and bias as separable aspects of causal learning, and suggests that some causal illusions may not really be “illusions” at all—they may simply reflect a bias to report causal relations.

A sports player wears a new pair of socks to a game that their team wins. Dubbing them “lucky socks,” the player subsequently wears the same pair of socks to every game, under the false impression that they can somehow influence the outcome of the game. Although humans display a remarkable sensitivity to causal relations from early childhood—as early as 6 months of age (e.g., Cohen & Oakes, 1993; Leslie, 1982, 1984; Leslie & Keeble, 1987; Oakes & Cohen, 1990; Saxe & Carey, 2006)—we are also susceptible to *illusions of causality*: Like the sports player, we often perceive that, and behave as if, unrelated events are causally related (Beck & Forstmeier, 2007; Blanco, 2017; Matute et al., 2015). Such causal illusions are thought to underlie superstitious and pseudoscientific beliefs and behaviours, such as wearing lucky socks to win a sports game, not walking under a ladder to avoid bad luck, or taking herbal pills to cure an illness (Matute et al., 2011, 2015; Torres et al., 2020).

Theories of causality generally posit three cues that organisms use to infer cause-and-effect relations: (1) *temporal order* (or *temporal priority*), that is, causes should precede the effects; (2) *contingency*, that is, causes and effects should co-occur; and (3) *contiguity*, that is, causally related events should occur close together in space and time (Hume, 1739/1888; see also Killeen, 1981; Young, 1995). According to this view, causal illusions arise because of experience with coincidental pairings between events. That is, if an apparent cause (e.g., sock-wearing) is coincidentally followed by an apparent outcome (e.g., winning a sports game), the two events may *appear* related. The more often this coincidental pairing is experienced, the stronger the apparent relation between them, and hence the stronger the illusion of causality (Blanco et al., 2011; Matute et al., 2015, 2019). In other words, as the name suggests, causal illusions arise because of failures to detect the absence of a causal relation between events.

In support of this *discriminability* view of causal illusions, a large body of research has shown that people are more likely to report a causal relation between two events if those events coincide more frequently and occur in closer temporal proximity. Shanks et al. (1989) asked participants to judge whether pressing a key caused a triangle to light up and found that participants reported a higher degree of control over producing the outcome when it followed a key press more frequently (i.e., the contingency was stronger), and when the delay between a key press and the outcome was shorter (i.e., stronger temporal contiguity; see also e.g., Elsner & Hommel, 2003; Greville et al., 2020; Greville & Buehner, 2010, 2016; Rudski, 2000; Shanks & Dickinson, 1987; Ward & Jenkins, 1965; Wasserman et al., 1983; Wasserman & Neunaber, 1986). In a similar procedure, Rudski (2000) showed that participants’ ability to detect a contingency between their behaviour and an outcome decreased when the outcome was more delayed. In addition, with longer behaviour-outcome delays, participants’ response patterns became more stereotyped—they repeated the same behavioural pattern in each trial, even though no such pattern was required to produce the outcome (see also e.g., Alloy & Abramson, 1979; Bloom et al., 2007; Catania & Cutts, 1963; Hayashi & Modico, 2019; Langer, 1975; Matute, 1995, 1996; Ono, 1987; Rey et al., 2020; Rudski et al., 1999; Sheehan et al., 2012; Wagner & Morris, 1987).

In addition to experience with adventitious event pairings, causal illusions may also reflect factors that bias participants to report (or not report) causal relations. Specifically, prior knowledge, expectations, or instructions can increase or decrease the likelihood of reporting causal relations between events. For example, illusions of causality are weaker when participants are pre-trained to expect the outcome to occur at a high base rate, compared with when they are pre-trained to expect the outcome to occur at a low rate (e.g., Blanco et al., 2020; Blanco & Matute, 2019; Buehner & May, 2004; Collins & Shanks, 2006; Vicente et al., 2023; Yarritu & Matute, 2015; Yarritu et al., 2015). Prior experiences of causality may also generalise, meaning that a causal relation (real or spurious) learned in one context may influence judgements of causality in similar contexts (Jin et al., 2022). Furthermore, organisms may have evolved mechanisms that are especially sensitive to detecting potential correlations between events, perhaps because such mechanisms are likely to facilitate faster learning and adaptation to environmental change (Abbott & Sherratt, 2011; Beck & Forstmeier, 2007; Foster & Kokko, 2009). Although this predisposition may give rise to causal illusions, such illusions may be less costly than failing to detect real cause-and-effect relations (consider, for instance, the consequences of failing to learn that rustling leaves overhead means that a predator is nearby, versus the consequences of making this assumption when it is not true).

Thus, illusions of causality may reflect failures of *discriminability*, coupled with a *bias* to detect causal relations. One way to separate discriminability and bias in causal learning scenarios is to conceptualise such scenarios as signal detection tasks, in which organisms detect the presence or absence of a causal relation between events (Allan et al., 2005, 2007, 2008; Perales et al., 2005; Siegel et al., 2009). According to Signal Detection Theory (SDT; Davison & Tustin, 1978; Green & Swets, 1966), performance in such tasks is characterised by two independent measures (Figure 1). *Discriminability* (or sensitivity) quantifies the organism’s ability to distinguish accurately between stimuli; in the context of causal learning, this refers to how easy it is to detect the presence or absence of a causal relation between events—the easier this is, the more accurate the causality detection, and hence the higher the discriminability. The second measure, *bias* (or criterion), reflects the threshold of evidence required for an organism to report the signal’s presence; the more liberal the criterion, the more often the organism will report a causal relation, whereas a strict criterion biases organisms to report the absence of a relation. To summarise, SDT effectively states that causal illusions arise (1) because of a genuine failure to detect that adventitiously paired events are not causally related, (2) because organisms are simply biased to behave as if causal relations exist, or (3) a combination of these.

**Figure 1.**
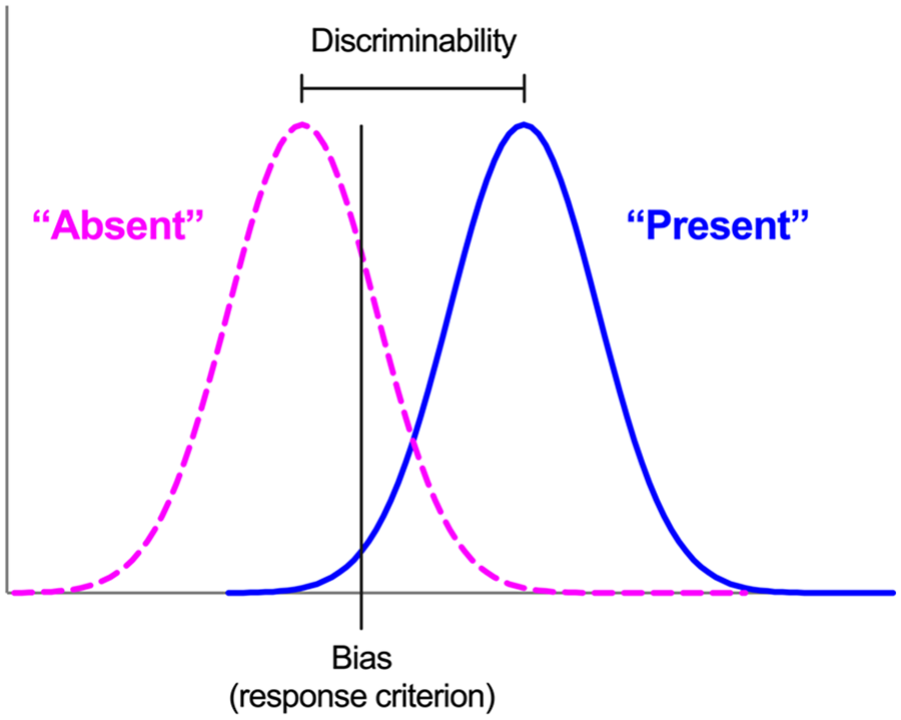
Signal Detection Theory. “Absent” and “Present” refer to the absence or presence, respectively, of a signal (i.e., of a causal relation, in the context of a causal learning task).

In one of the first studies to take an SDT approach to causal learning, Killeen (1978, 1981) tested whether pigeons could detect causal relations between their own behaviour and an outcome. In Killeen’s study, pigeons’ pecks to a centre key sometimes caused two side keys to illuminate, whereas at other times, the side keys illuminated independently of responding. The pigeons’ task was to indicate whether they had caused this event by choosing between two side keys, one corresponding to reporting “I did it” and the other to “I didn’t do it.” Accuracy was about 80% correct, suggesting that the pigeons could indeed discriminate whether or not they had caused the event. This discrimination appeared to depend on the delay between a response and the illumination of the side keys, with longer delays increasing the likelihood of reporting “I didn’t do it,” probably because the delay made it easier to detect that the event occurred independently of responding. In addition, the relative payoffs for reporting the presence of a causal relation influenced bias: The pigeons were more strongly biased to report “I did it” when the reinforcer magnitude for doing so was larger than reporting “I didn’t do it.” Killeen concluded that subjects can indeed discriminate the presence or absence of causal relations between their behaviour and subsequent events, but they may sometimes behave as if a causal relation exists because of factors (e.g., reinforcer magnitude) that bias them to do so (see also Killeen & Smith, 1984). Similarly, in a recent replication of Killeen’s experiment, we (Gomes-Ng et al., 2023) found that pigeons were only more likely to report that they had caused an event if they were inherently biased towards the key location associated with responding “I did it.”

Findings from the human contingency judgement literature corroborate those from the animal learning literature. Allan et al. (2005) asked participants to judge the extent to which a cue (a hypothetical chemical) caused an outcome (survival of a hypothetical bacterial strain) and found that participants were more likely to report a causal relation when the outcome occurred more frequently. SDT analyses indicated that this was because of changes in bias, but not discriminability; that is, outcome frequency influenced participants’ overall tendency to report a causal relation, but not their ability to detect such relations (see also Allan et al., 2007, 2008). Perales et al. (2005; see also Jozefowiez et al., 2022) obtained similar results when they manipulated cue frequencies, and they further showed that participants were more likely to report causal relations when the payoff for doing so was higher (i.e., when participants earned more points for correctly reporting the presence of a causal relation than for reporting the absence of a relation). Thus, like the work by Killeen (1978), these findings suggest that some causal illusions reflect biases, perhaps produced by high frequencies of potential causes or outcomes, and by relatively larger payoffs for reporting causal relations. In contrast, the nature of the contingency (positive or negative) may influence discriminability; Maia et al. (2018) found that participants were more sensitive to causal relations when asked to report whether a cue increased the probability of an outcome (a positive contingency) than when they reported whether a cue decreased outcome probability (a negative contingency). Taken together, these findings collectively lend support to a signal detection approach to causal learning and demonstrate the value of this approach for identifying the mechanisms underlying causal illusions.

At present, only a handful of studies have taken a signal detection approach to causal learning. Therefore, the current study also drew on a signal detection framework to investigate further causality detection in humans. Specifically, whereas previous studies with humans asked participants to judge the degree of causation between two external stimuli, we asked people to judge whether their own *behaviour* caused an event. This is an important distinction because many causal illusions—particularly those relating to superstition or pseudoscience—concern relationships between an individual’s behaviour (such as wearing lucky socks or taking herbal pills) and an apparent outcome of that behaviour, rather than between two external events. Hence, compared with past research that has focused on causality detection with external events, this study is more relevant to understanding the mechanisms underlying superstitious behaviour.

In addition, this experiment aimed to investigate the cross-species generality of the approach used to investigate causality detection in non-human animals (Gomes-Ng et al., 2023; Killeen, 1978; Killeen & Smith, 1984). To that end, we arranged a discrete trial procedure for humans that systematically replicated previous work with pigeons (Gomes-Ng et al., 2023). Participants’ responses to a stimulus were followed by point gain, point loss, or no change in points in some trials, whereas in other trials, these events occurred independently of responding. Participants then reported whether they had caused the event by choosing between two coloured circles, one corresponding to reporting “I did it” and the other to “I didn’t do it.” Thus, there was a weak positive contingency between behaviour and events, such that about half of the events in a session were contingent on a response and the other half were not contingent on responding. This meant that participants had roughly equal experience with response-dependent and response-independent events of each type. Given that unequal cue or outcome frequencies can bias responding in causal learning tasks (Allan et al., 2005, 2007, 2008; Jozefowiez et al., 2022; Perales et al., 2005; see also Baddeley & Colquhoun, 1969; Bedi et al., 2023; McCarthy & Davison, 1979), arranging equal experience with response-dependent and response-independent events ensured that performance measures were not influenced by unequal cause or outcome frequencies, and hence any differences in discriminability or bias between trials arranging point gain, point loss, and no change in points likely reflected the type of event (rather than other factors, e.g., experience, impacting on performance).

## Method

### Participants

We aimed to collect data from as many participants as possible within one academic semester (about 6 months). Our final sample consisted of 371 undergraduate students enrolled at a large university in New Zealand, who participated in one experimental session in exchange for course credit. Age and gender data were not recorded. Post hoc sensitivity power analysis with this sample size and α = .05 indicated 95% power to detect a minimum effect size of η^2^ = .007.

### Procedure

The experiment ran on PsyToolkit (Stoet, 2010, 2017). Before the session, participants gave written informed consent and then read the following instructions:

In this experiment, you will see a yellow circle on the screen. You can click on the circle using your computer mouse. Sometimes, your clicks will result in either no change in points, point gain, or point loss. Other times, these consequences will happen, but your clicks did not cause them.

You’ll then be given a choice between two different coloured circles. If you think that you caused the consequence, select the purple circle. If you think that you did not cause the consequence, select the orange circle. If you choose correctly, you’ll earn points! The more points you earn, the shorter the experiment will be.

The structure of each trial followed a 0-s delayed matching-to-sample (MTS) procedure. In MTS, a sample stimulus is presented for a short period of time, after which the sample is removed and comparison stimuli are presented immediately. One comparison stimulus “matches” the sample either identically or symbolically, and choice of the matching stimulus is reinforced while choice of any other (non-matching) stimulus is not. In this experiment, the sample stimulus was an event (point gain, point loss, or no change in points) which was delivered either *contingent* on the participant’s response or *independently* of responding. The comparison stimuli were two coloured circles (purple and orange), one of which corresponded to reporting “My response caused the event” and the other to “My response did not cause the event.” Thus, the key question was whether participants could detect whether or not the event delivery was contingent on a response. Hereafter, for brevity, we term trials in which the event delivery was contingent on responding as “response-dependent,” and trials in which the event was delivered independently of responding as “response-independent.” The experimental session lasted for 1 hr or until 240 trials had occurred, whichever occurred first. On average, participants completed 227.07 trials (*SEM* = 0.97) during the session.

At the beginning of each trial, the type of event (point gain, point loss, or no change; *p* = .33) and its delivery (response-dependent or response-independent; *p* = .50) were selected probabilistically. The mean percentage of trials of each type closely matched these arranged probabilities, with 33.3% (*SEM* = 0.07) of trials arranging point gain, 33.3% (*SEM* = 0.07) arranging point loss, and 33.4% (*SEM* = 0.07) arranging no change in points. For each of these event types, 50% (*SEM* = 0.1) were response-dependent.^1^ Trials were presented in a random order. The trial type (i.e., response-dependent or response-independent) was not signalled to participants; hence, the type of trial only became apparent to participants after the delivery of the response-dependent or response-independent event.

#### Sample phase

**Figure 2.**
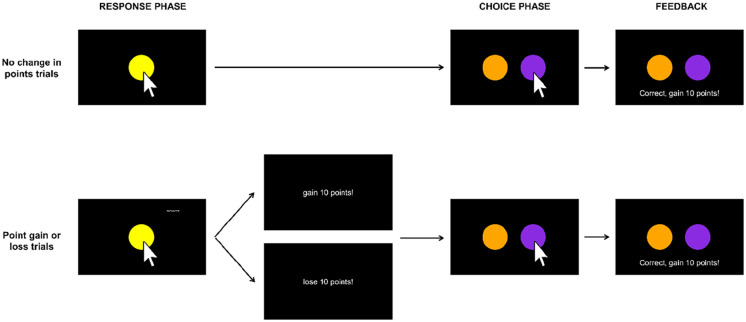
Trial structure. During the response phase, participants responded to a yellow circle. This was followed by a response-dependent or response-independent point gain or loss (“gain 10 points!” or “lose 10 points!” presented for 1.2 s), or no change in points. In the choice phase, choosing purple (displayed on the left in the diagram) was correct after response-dependent events and choosing orange (displayed on the right in the diagram) was correct after response-independent events. Participants received feedback (“Correct, gain 10 points!” or “Incorrect, lose 10 points!”) after choices. The feedback depicted above is for a correct response after a response-dependent event. See text for further details.

Figure 2 shows a diagram of the procedure. After the trial type was selected according to the procedure described above, the trial began with the presentation of a yellow circle in the centre of the screen (the *sample* phase). Participants responded to the circle by clicking it with their computer mouse. The circle remained on the screen for a randomly chosen duration ranging from 1 to 3 s, and during this time, participants could respond to the circle by clicking it with their computer mouse. Clicks had no effect during this time but were recorded by the experimental programme.

After the randomly chosen duration had elapsed, the event for that trial (point gain, point loss, or no points) was scheduled. In response-*independent* trials, the yellow circle was removed and the event was delivered. In response-*dependent* trials, the participant’s next response to the yellow circle resulted in its removal and the delivery of the event. Thus, in response-dependent trials, the programme waited for participants to make a response (as in a typical variable interval, VI, schedule) before delivering the event. If such a response never occurred, the programme continued waiting and eventually timed out after an hour (in reality, this never occurred as participants did respond). If the event was point gain or loss, the words “gain 10 points!” or “lose 10 points!,” respectively, were displayed in the centre of the screen for 1.2 s, and then two choice stimuli were presented. If no change in points was arranged, the choice stimuli were presented immediately.

#### Comparison (choice) phase

Our key question was whether could participants discriminate between an event delivered *contingent* on their responding and an event delivered *non-contingent* on responding. As such, in the comparison phase, participants answered this question by choosing between an orange and a purple circle, which were presented side-by-side in the centre of the screen (locations were counterbalanced across trials). In response-dependent trials, choosing the purple circle was correct, whereas in response-independent trials, choosing the orange circle was correct. Hence, the purple circle corresponded to “I did it” and the orange circle to “I didn’t do it.” After participants chose a circle by clicking it, they received feedback; the words “Correct, gain 10 points!” or “Incorrect, lose 10 points!” were displayed for 1.2 s below the choice stimuli, depending on whether they chose correctly or incorrectly. Hence, feedback was differentiated from the response-dependent or response-independent point gain or loss by the addition of the words “Correct” or “Incorrect.” Feedback was presented in every trial. After this feedback, the screen was cleared and a 1-s intertrial interval occurred.

A counter in the top-right of the screen kept track of total points during the session. The point incentive was arranged to motivate participants to respond. All study procedures were approved by The University of Auckland Human Participants Ethics Committee (ref. 024538).

## Results

### Acquisition

**Figure 3.**
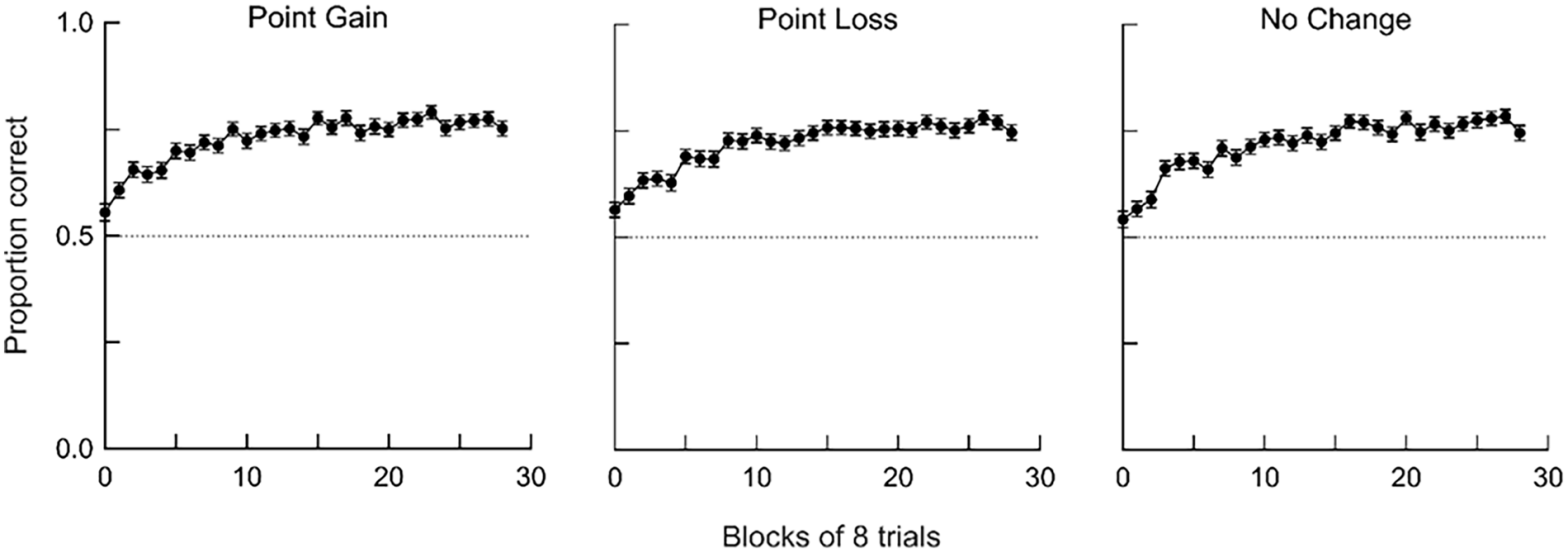
Changes in accuracy across the experimental session. Accuracy was calculated in blocks of eight trials. Data are averaged across participants, and error bars show the SEM.

We first assessed acquisition and changes in performance across experimental sessions. Correct and incorrect responses were separated based on trial type and aggregated into blocks of eight trials, and we used these response counts to calculate proportion correct in each block. Figure 3 shows mean (averaged across participants) accuracy across blocks for the three types of events (point gain, point loss, and no change in points). Proportion correct was close to chance (i.e., .5) at the beginning of the session, increased rapidly across successive blocks, and stabilised at about .80 by halfway through the session. Thus, participants quickly learned the contingencies. Because accuracy was stable in the last half of sessions, all subsequent analyses use data from the last half of sessions.

### Discriminability and bias

To separate participants’ ability to detect causal relations from any bias towards one response, we calculated independent measures of discriminability and bias (log *d* and log *b*, respectively; Davison & Nevin, 1999; Davison & Tustin, 1978) using the following formulae:

(1) $$log\;d=0.5\;log\left(\frac{B_{correct|dependent}}{B_{error|dependent}}.\frac{B_{correct|independent}}{B_{error|independent}}\right)$$

(2) $$log\;b=0.5\;log\left(\frac{B_{purple|dependent}}{B_{orange|dependent}}.\frac{B_{purple|independent}}{B_{orange|independent}}\right)$$

In Equation 1, $B_{x|y}$ represents the number of correct or incorrect (*x*) responses in trials in which the consequence was delivered response dependently or independently (*y*). More positive values of log *d* indicate more correct than incorrect responses (i.e., higher discriminability, and a greater ability to detect causal relations accurately), and a value of zero indicates chance performance. Equation 2 is similar, except that responses to the purple and orange circles (*x*) are used, and positive values indicate a bias to choose the purple circle (i.e., to report a response-*dependent* event) and negative values indicate a bias to choose the orange circle (i.e., to report a response-*independent* event). A log *b* value of zero indicates no systematic bias. We calculated log *d* and log *b* values for each participant. Because some participants made no correct or incorrect responses, which meant that some response counts were zero, we added .5 to all response counts for calculations of log *d* and log *b* (Hautus, 1995).

**Figure 4.**
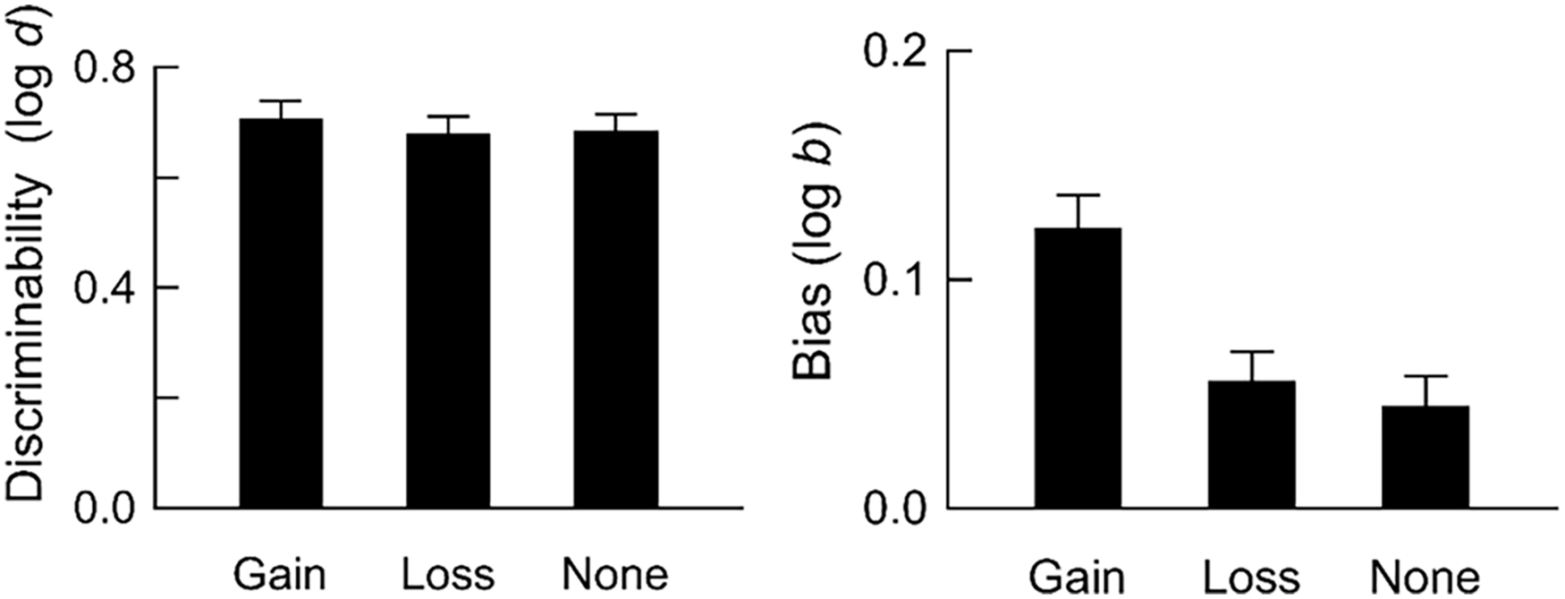
Discriminability and bias. The left panel shows discriminability (log *d*; Equation 1) and the right panel shows bias (log *b*; Equation 2). Data are averaged across participants. Error bars show the SEM.

Figure 4 shows mean discriminability and bias (Equations 1 and 2, respectively) for trials with point gains, point losses, and no change in points. Discriminability was slightly higher after point gains than after point losses or when no change in points occurred, but these differences were not statistically significant, *F*(2, 740) = 2.48, *p* = .085, η^2^ = .007. Hence, there were few systematic differences in participants’ ability to detect whether they had caused different types of events. In contrast, there were clear differences in bias between events: Participants were more likely to report that they had caused point gains, compared with point losses and no change in points, *F*(1.96, 725.91) = 14.61, *p* < .001, η^2^ = .038.

### Effects of response rates on accuracy in response-independent trials

Participants’ response rates to the yellow circle (see Figure 2; hereafter, *response rates*) may have impacted their ability to detect whether they caused the event. Specifically, the higher the response rate, the more likely it was that a response would have coincided with a response-independent event, and this may have made it harder to discriminate the cause of that event. We thus assessed the relationship between responding and accuracy (measured as proportion correct) in response-independent trials. We quantified responding in response-independent trials in two ways: (1) response rates, which were calculated by taking the inverse of the average time (in ms) between responses to the yellow circle and then multiplying by 1,000 to yield responses per second; and (2) proportion of response-independent trials in which participants made at least one response.

**Figure 5.**
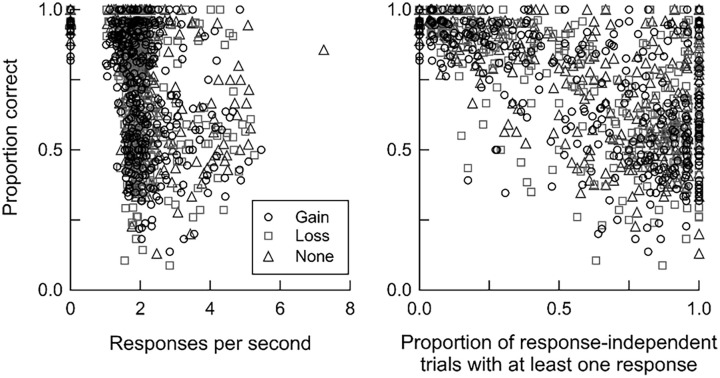
Relationship between accuracy and responding in response-independent trials. Responding in response-independent trials was measured as responses per second (left panel) or as the proportion of trials with at least one response (right panel). Circles, squares, and triangles show data from trials with point gains, point losses, and no change in points, respectively.

Figure 5 plots proportion correct in response-independent trials as a function of overall response rates to the yellow circle for individual participants, or as a function of the proportion of response-independent trials with at least one response. Most participants responded at a rate of 2 responses per second (*M* = 1.94, *SD* = 0.04). There was considerable variability in accuracy, particularly when the proportion of trials in which participants made at least one response was higher. Nevertheless, in general, accuracy in response-independent trials was lower when response rates were higher, *r*(369) = −.45, −.44, and −.25 for point gain, point loss, and no change in points, respectively (all *p*s < .001). This negative relationship was more pronounced when we measured responding using the proportion of trials in which participants made at least one response, *r*(369) = −.64, −.65, and −.66 for point gain, point loss, and no change, respectively (all *p*s < .001). Therefore, overall, the more a participant responded in response-independent trials, the more likely they were to report incorrectly that events depended on their own responding.

### High- versus low-performing participants

Inspection of individual participants’ data indicated that some participants were better at detecting causal relations than others. Therefore, our final analysis separated participants based on their level of performance to examine any potential differences in discriminability and bias between high- and low-performing participants. Participants for whom proportion correct exceeded .70 in all trial types were grouped together as “high performers” (*N* = 187), and all other participants were grouped together as “low performers” (*N* = 184). Grouping participants with this criterion ensured that high performers could discriminate relatively accurately across all six trial types, and that participants with strong biases (which would have resulted in high accuracy in some, but not all, trials) were not classified as high performers.

**Figure 6.**
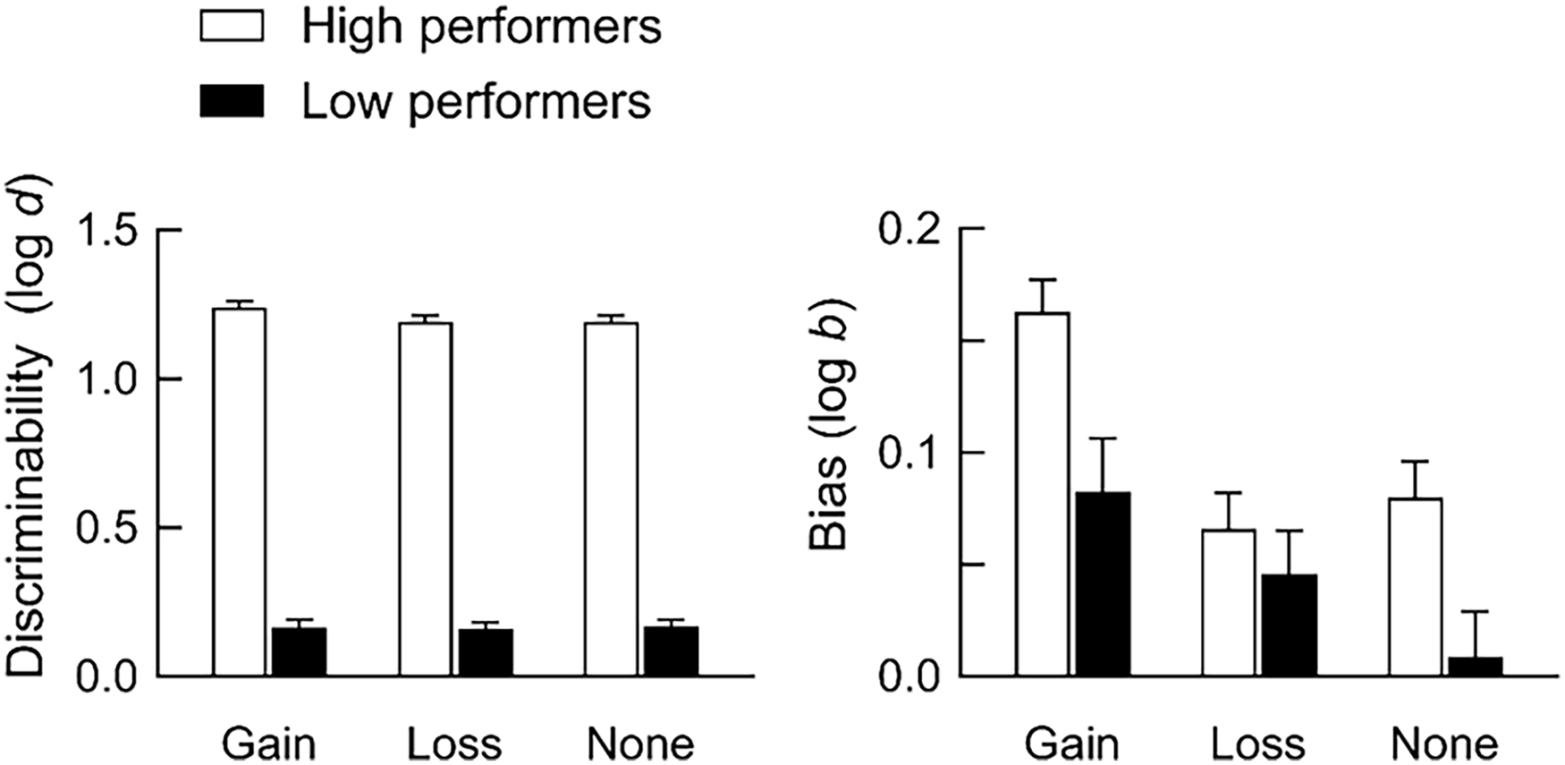
Discriminability and bias for high and low performers. The left panel shows discriminability (log *d*, Equation 1) and the right panel shows bias (log *b*, Equation 2). Unfilled bars show data for high performers, and filled bars show data for low performers. Data are averaged across participants in each group, and error bars show the SEM.

Figure 6 shows discriminability and bias for high and low performers. Both discriminability and bias were higher for high-performing participants than for low-performing participants. For high performers, discriminability was higher after point gains than after no change in points, *F*(2, 372) = 3.72, *p* = .025, η^2^ = .020. In contrast, discriminability was similarly low across events for low performers, *F*(2, 366) = 0.15, *p* = .857, η^2^ < .001. Both high- and low-performing participants were more strongly biased to report that point gains depended on their responding, *F*(2, 327) = 12.39, *p* < .001, η^2^ = .062 for high performers, *F*(1.89, 346.60) = 5.03, *p* = .008, η^2^ = .027 for low performers. Hence, even when participants could discriminate relatively accurately whether they had caused an event (i.e., high performers), they were still biased to attribute events to their own behaviour, particularly when those events were appetitive.

### Summary of main results

Mean accuracy was close to 80% correct by the end of experimental sessions, indicating that participants could detect whether they had or had not caused point gains, point losses, or no change in points (Figures 3 and 4). Overall, there were few differences in discriminability between events, whereas bias to report that events depended on responding was stronger after point gains than after other events. Such bias was also stronger when response rates were higher in response-independent trials (Figure 5) and for higher-performing participants (Figure 6).

## Discussion

Humans often behave as if spurious relations exist between unrelated events (Blanco, 2017; Matute et al., 2015). Such *superstitious* behaviour may reflect failures of discrimination—a genuine inability to detect that causal relations do not actually exist—and general biases to report or behave as if events are causally related (Killeen, 1978, 1981). Here, we took a signal detection approach to investigate the mechanisms underlying such causal illusions. Participants reported whether their behaviour (a computer mouse click) caused a point gain, a point loss, or no change in points by responding to two arbitrary coloured circles. Accuracy was relatively high, indicating that participants could indeed detect whether events were dependent on or independent of their responding. Our most notable finding was that as such discriminability increased, bias to report that events depended on responding *strengthened*, particularly when those events were desirable (point gains). Overall, these findings suggest that causal illusions may sometimes arise because of biases, rather than due to genuine failures of discrimination. Thus, this study highlights discriminability and bias as independent aspects of causal learning.

Previous research has shown that the frequency of events, and the relative payoffs for reporting (or not reporting) causal relations, impact bias but not discriminability in contingency judgement tasks (e.g., Allan et al., 2005; Jozefowiez et al., 2022; Killeen, 1978; Killeen & Smith, 1984; Maia et al., 2018; Siegel et al., 2009). That is, these variables push participants to report the presence of causal relations more (or less) frequently by producing more liberal (or stricter) decision criterion, independently of participants’ ability to detect whether causal relations exist. Our findings add to this body of research by suggesting that the *type* of event also impacts bias: There were few differences in discriminability between events, whereas participants were more strongly biased to report that they had caused point gains compared with trials in which they lost points or there was no change in points. This result is unlikely to reflect differences in experience with different trial types or differences in response rates because participants had equal experience with all trial types and response rates were similar across trial types.

Why might humans be more likely to attribute appetitive events, compared with aversive events, to their own behaviour? Rudski et al. (1999) hypothesised that heightened sensitivity to punishment contingencies, implying greater accuracy at detecting causality, may explain why causal illusions are weaker when participants lose compared with when they gain points. Our findings appear inconsistent with this hypothesis because we found no such differences in sensitivity (i.e., discriminability) between events. An alternative explanation is that people attribute desirable outcomes to their own behaviour and undesirable outcomes to external forces because this “self-serving” bias (Bradley, 1978; Miller & Ross, 1975) protects self-esteem and promotes psychological wellbeing. Research examining self-serving biases has produced mixed results, with some supporting such biases (e.g., Allen et al., 2020; Alloy & Abramson, 1979; Alloy & Clements, 1992; Beckman, 1970; Brewin & Shapiro, 1984; Taylor & Brown, 1988) and other studies suggesting that self-serving biases are procedural artefacts (e.g., Colvin & Block, 1994; Duval & Silvia, 2002; Miller & Ross, 1975; Yarritu et al., 2014). Although this study did not examine self-serving biases per se, our findings appear to lend support to the hypothesis that people are biased to attribute desirable, but not undesirable, events to their own behaviour: Our participants were more strongly biased to report response-dependent events after point gains than after point losses, even though the frequency of the cause (i.e., responding) was roughly equal between events.

More generally, the notion that appetitive and aversive events have differential effects on behaviour is not new. For example, Kahneman and Tversky’s (1979) prospect theory suggests that people behave differently when choosing between options framed in terms of gains than between options framed in terms of losses. To illustrate, when presented with a choice between probabilistic and certain gains (e.g., 50% chance of winning US$1,000 vs 100% chance of winning US$400), people tend to prefer the certain option; in contrast, if the choice is between probabilistic and certain losses, people prefer the probabilistic option. This is thought to occur because the effects of losses are disproportionately larger than the effects of equal-sized gains; as a result, humans are more likely to take risks when faced with the prospect of a loss compared with the prospect of a gain. Along similar lines, Rasmussen and Newland (2008) showed that the effects of money loss on human choice were three times greater than the effect of equal-sized money gain. Other studies have shown that reinforcer and punisher effects differ qualitatively (e.g., Gonçalves & Silva, 2015; Kubanek et al., 2015; Lie, 2010), and that different patterns of neural activity underlie reinforcement and punishment learning (Kim et al., 2015). Although our study differs in its approach from this prior research, the present findings and those of past studies collectively suggest that appetitive and aversive outcomes have fundamentally different effects on behaviour.

In the present procedure, participants were given explicit instructions stating that events were either dependent on or independent of their responding, and that they should choose the purple circle after response-dependent events and the orange circle after response-independent events. Despite these instructions, almost half (49.6%) of the participants were low performers, and of these, most (*N* = 107) did not meet the performance threshold of 70% correct in *any* trial type. One reason for such low performance is that these participants may not have been sufficiently motivated to respond correctly because points had no monetary value. Alternatively, these participants may not have attended to or understood the instructions. Furthermore, even with instructions, behaviour was probably also controlled by experienced contingencies during the session: Participants needed to discriminate between response-dependent and response-independent events, and to learn from corrective feedback during the choice phase. To the extent that instructional control was weaker, performance would have depended more strongly on participants’ ability to discriminate, and learn from, these contingencies. Low-performing participants may have been less sensitive to, and hence their behaviour less strongly controlled by, contingencies compared with high performers (see e.g., Fox & Kyonka, 2017; Galizio, 1979; Hayes, Brownstein, Haas & Greenway, 1986; Hayes, Brownstein, Zettle, et al., 1986; Kaufman et al., 1966; Podlesnik & Chase, 2006; Shimoff et al., 1986 for more on human contingency learning). Indeed, the results of a smaller, unpublished version of the present experiment suggested that in the absence of instructions, the contingencies were difficult for participants to discriminate, resulting in poor performance akin to low performers in the present experiment. Therefore, individual differences in performance may reflect a combination of differences in motivation to respond correctly, the strength of instructional control, and sensitivity to contingencies.

Two aspects of the present procedure may have contributed to low performance for some participants. First, the choice stimuli were presented immediately after the offset of the yellow circle in trials with no change in points, whereas point gain or loss was interpolated between the offset of the yellow circle and choice stimuli in trials arranging point gains or losses. This difference may have made the contingencies less clear in no-change trials, in which no obvious “event” occurred. Second, the contingencies leading to point gain or loss may have been somewhat obscure because they differed between phases—participants gained or lost 10 points dependent on or independent of responding to the yellow circle, and then after correct or incorrect responses in the choice phase (Figure 2). Although these two procedural aspects may have made the task contingencies more difficult to discriminate, it is worth noting that discriminability was similar across trial types, suggesting that the effects of these aspects were relatively small. To be sure, making the contingencies clearer (e.g., arranging an event, such as “no change in points” in no-change trials, and further differentiating response-dependent or -independent point gains or losses from corrective feedback in the choice phase) and exploring the effects of individual differences (e.g., sensitivity to contingencies, attention to instructions, susceptibility to superstitious or paranormal beliefs) on performance in future research may help to identify the variables characterising high- versus low-performing participants.

What does this study suggest about the mechanisms underlying causality detection? In general, the dominant view is that associative-learning mechanisms underlie causal learning. According to this view, organisms learn that events are causally related through experiencing repeated pairings (real or adventitious) of those events (Blanco et al., 2011; Matute et al., 2015, 2019). The implication of this explanation is that causal illusions arise because organisms fail to discriminate that events are independent. Consistent with this explanation, our participants were more likely to report a spurious relation between their behaviour and a response-independent event if they had responded in response-independent trials (Figure 5). However, we also found that participants were more strongly biased to report that events depended on responding when discriminability was *higher* (Figures 4 and 6)—a result that is inconsistent with the associative-learning perspective that causal illusions arise because of failures of discrimination.

It is important to note that our response measures were proxy measures of response-event pairings because such pairings depend on *when* participants respond relative to the event. Thus, responding in response-independent trials may not necessarily result in more coincidental pairings if those responses are not temporally contiguous with the event. A limitation of the current study is that we were unable to analyse the time between participants’ responses and events due to the nature of the data collected. Hence, our conclusions about adventitious event pairings are presently tentative. Nevertheless, the negative relationships between responding and discriminability here certainly lend some support, and our conclusions are largely consistent with similar research suggesting that the delay between a behaviour and a subsequent outcome, and factors that influence bias (e.g., relative reinforcer rates) contribute to causal illusions (e.g., Gomes-Ng et al., 2023; Killeen, 1978; Killeen & Smith, 1984). To be sure, however, a replication of the current study should also include measures of the time between participants’ responses and events, and could vary the temporal contiguity between responses and events (e.g., inserting a delay between responses and response-dependent events) to clarify how adventitious behaviour-event pairings impact participants’ ability to discriminate causal relations.

The current study is the first to replicate work using non-human animals (Gomes-Ng et al., 2023; Killeen, 1978; Killeen & Smith, 1984) in humans. Thus, the extent to which our results—and in particular, the differences in bias between appetitive and aversive events—are replicable remains to be seen. More generally, the SDT approach provides a way to determine the mechanisms by which different manipulations impact the strength of causal illusions. For example, do educational interventions reduce causal illusions (Barberia et al., 2013, 2018) because they increase participants’ ability to detect accurately causal relations, or because they shift their decision criterion for reporting causal relations? Relatedly, a handful of studies have shown that causal illusions are stronger under conditions of negative reinforcement (i.e., removal of an aversive event) than positive reinforcement (i.e., delivery of an appetitive event; Aeschleman et al., 2003; Bloom et al., 2007; Hayashi & Modico, 2019). SDT would help to clarify whether differences in discriminability or bias underlie these differences between the detection of positive and negative contingencies.

Finally, most studies of causality detection in humans use survey methods to measure causal illusions (e.g., Hayashi & Modico, 2019; Rudski, 2000; Rudski et al., 1999). Such methods may be disadvantageous because the wording of questions may skew participants’ responses to exaggerate or underestimate causal relations (e.g., Collins & Shanks, 2006), and self-report measures may not accurately represent behaviour (see Dang et al., 2020, for discussion). The extent to which self-report measures reflect the same processes as behavioural measures (i.e., discriminability and bias) is an open question. To address this, future studies should take both self-report and behavioural measures, and compare these to ascertain the extent of agreement between them.

In summary, the present findings demonstrate that people can judge causality relatively well, but they are still biased to attribute events to their own behaviour, particularly if those events are more desirable. Thus, when participants falsely report a causal relation in a contingency judgement task, researchers should not assume that this reflects a failure to detect the absence of a causal relation. Instead, such performance may reflect a bias (perhaps produced by differential reinforcement and inherent predispositions) to report causal relations. To be sure, separate measures of discriminability and bias should be taken, which will provide a clearer picture of the mechanisms underlying causal learning.

## Supplemental Material

sj-csv-1-qjp-10.1177_17470218241293418 – Supplemental material for When is a causal illusion an illusion? Separating discriminability and bias in human contingency judgements Supplemental material, sj-csv-1-qjp-10.1177_17470218241293418 for When is a causal illusion an illusion? Separating discriminability and bias in human contingency judgements by Stephanie Gomes-Ng, Sarah Cowie and Douglas Elliffe in Quarterly Journal of Experimental Psychology

## Notes

1. Expressed in terms of the number of trials, the mean number of response-dependent trials was 37.7 for point gain, 37.8 for point loss, and 37.9 for no change in points (all SEMs = 0.2). Likewise, the mean number of response-independent trials was 37.8 for point gain, 37.9 for point loss, and 38.1 for no change (all SEMs = 0.2)
